# Enhanced Coating Protection of C-Steel Using Polystyrene Clay Nanocomposite Impregnated with Inhibitors

**DOI:** 10.3390/polym15020372

**Published:** 2023-01-10

**Authors:** Aljawharah M. Alangari, Layla A. Al Juhaiman, Waffa K. Mekhamer

**Affiliations:** 1 Chemistry Department, King Saud University, Riyadh 12372, Saudi Arabia; 2 Department of Material Science, Institute of Graduate Studies, Alexandria University, Alexandria 5422004, Egypt

**Keywords:** carbon steel, local clay, polystyrene, polymer clay nanocomposites, microcapsules, enhanced coating protection

## Abstract

Polymer–Clay Nanocomposite (PCN) coatings were prepared using the solution intercalation method. The raw Khulays clay was treated with NaCl to produce sodium clay (NaC). Thereafter, Cetyl Pyridinium Chloride (CPC) was used to convert NaC into the organic clay form (OC). PCN was prepared by adding polystyrene as the matrix to different weights of OC to prepare 1 wt.% and 3 wt.% PCN. To enhance the coating protection of C-steel in NaCl solution, PCN coatings were added to microcapsules loaded with some corrosion inhibitors PCN (MC). The microcapsules are prepared by the encapsulation of rare-earth metal Ce^+3^ ions and Isobutyl silanol into polystyrene via the Double Emulsion Solvent Evaporation (DESE) technique. Characterization techniques such as FTIR, X-Ray Diffraction (XRD), and Transmission Electron Microscopy (TEM) were employed. FTIR confirmed the success of the preparation, while XRD and TEM revealed an intercalated structure of 1 wt.% PCN while 3 wt.% PCN has a fully exfoliated structure. Electrochemical Impedance Spectroscopy (EIS), Electrochemical Frequency Modulation (EFM), and Potentiodynamic Polarization showed an enhanced protection efficiency of PCN (MC) coatings. The results demonstrated that the corrosion resistance (R_Corr_) of 3% PCN (MC) coating was higher than all the formulations. These PCN (MC) coatings may provide corrosion protection for C-steel pipes in many industrial applications.

## 1. Introduction

Steel and its alloys are widely used in many industrial applications, like the oil industry and engineering structures, due to their high strength and ductility. Carbon steel (C-steel) pipes are mainly used in a corrosive medium where corrosion prevention is important. Corrosion of steel alloys may lead to degeneration in their properties, waste of resources, safety, and environmental problems [1]. Therefore, protecting steel from corrosion has acquired high importance in minimizing economic losses, cost reduction, and achieving acceptable performances. Thus, a new generation of high-performance protective coatings that can provide long-term protection is required [2]. The idea behind the application of different corrosion control strategies lies in the removal of an electrochemical cell component, such as the cathode, electron-conductive path, corrosive environment, or even by modifying the metal to be protected [1,2]. Protective coatings are one of the best strategies in corrosion protection. Through protective coatings, corrosion can be controlled by one or more of these mechanisms: barrier property, cathodic protection, and inhibition, including the principle of anodic protection. Coatings consist of major components, including binders, pigments, solvents, and additives as inhibitors, surface-active agents, thickeners, and coupling agents. Corrosive components, such as ions, oxygen, and water, can penetrate the coating to reach the interface between the metal and that coating, causing a hidden corrosion process in the metal. Nanotechnology, including nanocomposites, contributed significantly to enhancing anticorrosive coating efficiency. Various types of nanofillers have been used for the preparation of these nanocomposites. Recently, clay got the attention of many researchers because of its ability to give a stunning enhancement to composite coating efficiency. It is also eco-friendly and naturally abundant [2]. Among all the well-known silicate types, layered materials like smectite clay, such as bentonite and montmorillonite (MMT), play an important role in the preparation of different novel polymer–clay nanocomposites (PCNs), since it has various properties, including a huge cationic exchange capacity (CEC) [3]. 

Bentonite clay minerals are naturally formed phyllosilicates consisting mostly of montmorillonite with (2:1) layer composition. A single layer consists of two tetrahedral sheets (Silica, SiO_4_), with one octahedral sheet (Alumina, Al_2_O_3_) sandwiched between them and can be expressed as (TOT). The gap between the layers is known as the interlayer or gallery or (d_001_ spacing), which can vary according to the cations present. The use of polymer nanocomposites mainly depends on their polymer and fillers, whether they are synthetic or natural with at least one dimension in a nanometer size scale, and they produce unusual properties that were not observed when using the same components separately [2]. 

Polymer clay nanocomposites (PCNs) have outstanding chemical, physical, mechanical, thermal, and barrier properties compared to polymers or conventional composites. PCN forms a maze-like pathway that retards the diffusion of various corrosive components. Protective coating prevents corrosive ions such as chloride, protons, or molecules like water and oxygen from penetrating through the film into the metal surface. However, resistance does not last for long, and it will be penetrated eventually, creating an internal pathway through which corrosive substances pass to the metal surface, causing corrosion of the metal [4,5,6,7,8,9,10,11,12,13]. Despite the high efficiency of organic and PCN protective coatings, long-term protection is not yet guaranteed. PCN form a rigid barrier, but once corrosive components permeate them, their properties begin to collapse, leading to corrosion at the interface between the metal and the corrosive medium [1,2].

Some previous studies highlighted that the organic coating could work as a reservoir for carrying corrosion inhibitors, which then promote the coatings’ life [14,15,16]. Currently, several studies have been conducted to develop protective coatings impregnated, loaded, or doped with some eco-friendly new additives called “green corrosion inhibitors”. These impregnated coatings not only provide enhanced barrier properties but also interact with the environment and protect the active sites on the metal surface whenever the corrosive components penetrate them. Recently, many studies investigating how the functionality and life of the coating can be improved have been carried out [14,15,16]. Publication on this line of study, i.e., smart coatings, has increased approximately tenfold from 2000–2010. Several criteria, including the type of material, type of polymer, functionality, applications, as well as stimuli (or triggers), can be used to classify smart coatings. There are many triggering mechanisms, including chemical mechanisms such as pH, humidity change, external triggers like UV radiation, temperature, a mechanical mechanism, and electrochemical potential. Smart coatings have a response to the surrounding environment, which in turn provides long-term protection through different—self-healing or self-repairing mechanisms, and the healing process may have more than one triggering mechanism associated with each other; for example, a chemical–mechanical mechanism.

Not long ago, smart anticorrosive coatings used were based on chromium compounds, which are known for their high toxicity, prompting many researchers to develop alternative coatings using ceramics, metals, or composites [14,15]. Often, smart coatings contain cerium, sodium salt of tetra thiomolybdate (VI), and organofluorine and organosilane compounds. Organosilane compounds are widely used in smart coatings as coupling agents since they can present various functionalities, including the inhibition of active corrosion sites on the metal substrate and the self-healing of the coating resin. The general formula of organosilanes is R–(CH_2_)–Si–X_3_, which include a silicon atom, a hydrolyzable alkoxy group (X), and a non-hydrolyzable group (R), such as amino, epoxy, vinyl, and methacrylate. 

Microcapsules (MCs) play an important role in smart protective coatings. These capsules are produced on a micrometer scale, the commercial ones usually range from 3–800 µm, and they consist of two main parts; a loaded material to be coated, which can be a solid, liquid or gas (known as Core) and an inert solid outer material that encapsulates the core (Shell) [5,14,15,16]. There are several techniques for encapsulation. The employed technique will depend on some factors, such as the hydrophobicity and hydrophilicity of polymers and loaded corrosion inhibitors, the self-healing mechanism, the matrix in the coating formulation, and the nature of the polymer used as a shell.

In our previous study [13], polystyrene clay nanocomposite was prepared using Khulays organoclay (OC). Differential Scanning Calorimetry (DSC) was applied for 1–10% PCN. The glass transition temperature decreased from 98.14 °C for pure PS to lower values for 1–3% PCN, then increased for 5% PCN to reach a higher Tg of 104.22 °C for 10% PCN. The exfoliated structure of the low clay-loading (1–3%) PCN leads to the movement of the polymer chains between the organoclay layers, and the PCN film becomes ductile and does not break when used as a coating material. In another study of our research group [6], we used 1, 3, 5, and 10% PCN as a protective coating of the C-steel. It was observed that the extent of protection provided by PCN coatings depended upon the loading of the organoclay, as found by other researchers [2,4,8]. At a higher clay loading than 3% PCN, the corrosion protection performance decreased, as proved by the electrochemical data [6].

In the present study, we will prepare the PCN with low clay loading at 1 and 3% PCN to obtain a PCN with an exfoliated structure to decrease the coating permeability. Moreover, it will improve the coating barrier properties explained by the concept of tortuous paths where the permeating molecules are forced to follow a wiggle path which will improve the metal coating protection. In order to enhance the protective properties of these PCN coatings, we aim to prepare a polystyrene/organoclay nanocomposite impregnated with an inhibitor prepared by double-emulsion solvent evaporation (DESE). In this study, several characterization techniques are applied, such as FT-IR, XRD, SEM, and TEM. Evaluation of the protection efficiency of C-steel in NaCl solution was done by performing three electrochemical methods, which are Electrochemical Impedance Spectroscopy (EIS), Electrochemical Frequency Modulation (EFM), and Potentiodynamic Polarization.

## 2. Materials and Methods

### 2.1. Materials

The raw clay (RC) was collected by a certified geologist from Khulays region, Jeddah, Saudi Arabia. It was previously found that this RC belongs to bentonite clay minerals [13]. The RC contains montmorillonite (35.22%), kaolinite (13.33%), mica (22.80%), quartz (8.57%), feldspars (6.66%), ilmenite (5.71), dolomite (3.81%), and gypsum (3.81%). The exact composition of this clay mineral was indicated by X-ray Fluorescence (XRF) [13] to be:

Na_0.67_K_0.13_Ca_0.02_Ba_0.04_(Si_7.47_Al_0.53_) (Al_2.59_Fe_0.78_Ti_0.14_Mg_0.44_Cr_0.04_) O_20_(OH)_4_

A local polystyrene ((M = 259,000 g/mol) was used from the SABIC company in Saudi Arabia. Carbon steel (C-steel) rods were provided by ODS Co., Schleswig-Holstein, Germany. To ensure the exposed cross-sectional area remained constant, the outer wall of the C-steel rod was covered by an epoxy resin. The chemical composition of the C-steel is shown in Table 1. The materials are summarized in Table 2.

### 2.2. Clay Separation, Saturation, and Organic Modification

Most previous studies used pretreated clay (obtained from the market) in the preparation of polymer clay nanocomposites. In our lab, we ground the local Khulays clay from the raw rocks to fine grain size in the micrometer range, as discussed in [13]. Our strategy of preparing PCN started with the modification of RC. Fifty grams of RC was dispersed in 500 mL of distilled water and shaken for two hours, then left overnight. The raw clay was centrifuged and then washed with distilled water, and this process was repeated for three days to remove any impurities in RC. Then, 300 mL of 0.5 M NaCl solution was added to the clay, and the suspension was mixed by shaking for 2 h and then left overnight. This process was repeated three times to produce sodium clay NaC. The cationic surfactant Cetyl pyridinium Chloride (CPC) was used in the ion exchange treatment to convert the NaC into the organic clay form (OC). It is known that the highest cation exchange capacity (CEC) of clay is about 85 meq/100 g [3]. Five grams of NaC was dispersed in 500 mL of distilled water. To ensure that the organic cations in CPC replace all the cations in the NaC structure, the amount of CPC corresponding to two times the CEC of clay was dissolved in 100 mL of distilled water. The CPC solution was added to the NaC suspension at a slow rate, and the mixture was stirred for 24 h at room temperature. The resulting organoclay (OC) was separated by centrifugation and washed with distilled water several times until no chloride ions were detected by 0.1 M AgNO3 solution. Finally, the OC was dried at 60 °C for 24 h in the oven.

### 2.3. Polystyrene-OC Nanocomposites (PCNs) Preparation

Anticorrosive coatings for C-steel based on polystyrene/organoclay nanocomposite (PCN) were successfully prepared using polystyrene (PS) as a matrix with different percentage weights of OC to prepare 1% PCN and 3% PCN, respectively [6,13]. In a 50 mL flask, a certain mass of OC (0.02 and 0.06 g) was added to 10 mL of toluene, and this suspension was stirred magnetically overnight at room temperature. Afterward, 2 g of PS was added, and the mixture was magnetically stirred for 6 h and then in an ultrasonic machine for 10 min. For characterization purposes, the PCN solution was cast into a Petri dish to allow the toluene to evaporate. The coating adhesion test was performed for the interface between C-steel and PCN coatings using a cross-hatch cutter instrument (Sheen Instruments, Cambridge, UK) according to ASTM D-3359-02.

### 2.4. Polystyrene Microcapsules (MCs) Preparation

Polystyrene microcapsules (MCs) loaded with Isobutylsilanol and Ce^+3^ ions as corrosion inhibitors were successfully prepared with the Double-Emulsion Solvent Evaporation (DESE) method following the procedures from a previous report [5]. The preparation included three steps, namely, the preparation of the aqueous inhibitor solution, first emulsion, and double emulsion labeled (W_1_), (W_1_/O), and (W_1_/O/W_2_), respectively. First, in a 50 mL flask, equal volumes of distilled water and 96 wt.% ethanol were added, and the pH was adjusted to pH 5 with glacial acetic acid. Cerium ions from Ce(NO_3_)_3_.6H_2_O salt were added to the solution with a concentration of 5000 ppm. Subsequently, 4 wt.% of isobutyl trimethoxy silane (IBTMS) was added. The solution was kept under magnetic stirring for 24 h to complete the hydrolysis process of the W_1_ solution. Second, in another flask, 2 g of PS was dissolved in 10 mL Dichloromethane (DCM), and 0.5 g of CPC was added as a cationic surfactant. Approximately 1 mL of W_1_ solution was added to the PS mixture under sonication for 2 min, resulting in a W_1_/O emulsion. Third, in a 200 mL flask, about 30 mL of 1 wt.% of Polyvinyl Alcohol (PVA) aqueous solution was added. Then, under magnetic stirring (300 rpm), W_1_/O was slowly added to the PVA solution. The last step of W_1_/O/W_2_ preparation was the addition of a large amount of 0.3 wt.% PVA aqueous solution. The double emulsion was left for 24 h under magnetic stirring for solvent evaporation. After that time, polystyrene microcapsules (MC) were filtered with a Büchner funnel, and the MC was washed with deionized water several times. Finally, MC appeared as a fine white powder, easily collected in a crucible and kept in a desiccator. The Ce^+3^ ions played the role of corrosion inhibitor and healing agent, whereas Silanol (Si–OH) was used as a corrosion inhibitor and a coupling agent for MC.

### 2.5. Coating the C-Steel Surface

The C-steel rods have an exposed surface area of 4.75 cm^2^, which was calculated using a Mitutoyo gauging tool. These rods went through a polishing process using abrasive papers of various grades 80, 220, 600, and 1000 for the purpose of scraping the surface and removing possible deposits and impurities. Thereafter, they were washed with distilled water, followed by immersion in acetone using an ultrasonic bath for 2 min to ensure the removal of any leftover impurities. Next, the C-steel rods were dried and left aside. There are three types of coating: the first one is PS coatings, the second one is PCN coatings, and third one is PCN (MC). To prepare the PS-coating solution, 2 g of PS was dissolved in 10 mL toluene and was stirred magnetically overnight at room temperature. For casting, the PCN solution or PS solution was applied dropwise on the C-steel as the first layer. A similar application was conducted on the second layer. It is important to note that each layer was left to air-dry for about 4 h, and subsequently dried in a Carbolite furnace for 24 h at 60 °C.

**Scheme 1:**
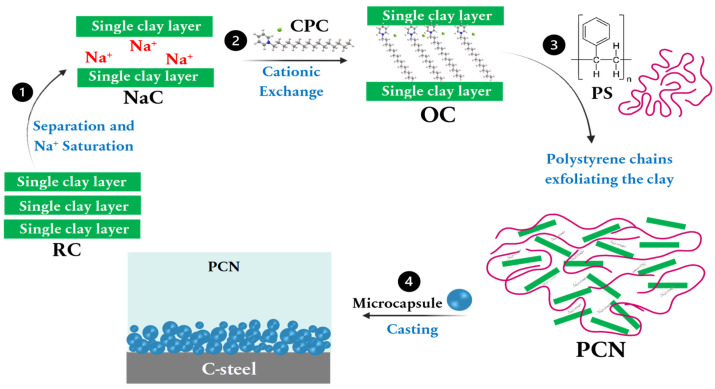
Polystyrene–organoclay nanocomposite synthesis and the coating of C-steel.

In the case of the new PCN coating impregnated with MCs, the suspended MCs in isopropyl alcohol were distributed on the surface of the C-steel rod and then left under atmospheric air for about 10 min until the alcohol evaporated. Next, the casting of the PCN solution was done following the procedure described previously. Finally, before performing electrochemical measurements, the thickness of the dry-casted coating on the electrode was determined using Elcometer 456 coating thickness gauge, and it was noted that the total thickness was in the range of 100 ± 15 µm. The synthesis of polystyrene–organoclay nanocomposite and the coating of the C-steel is depicted in Scheme 1.

### 2.6. Fourier Transform Infrared Spectroscopy (FT-IR)

The FT-IR was applied in the Department of Chemistry, College of Science, King Saud University. This technique was used to verify the success of the modification process done on RC by sodium chloride and the organic modifier CPC, as well as PCNs coatings and the MCs. The analysis was performed for all samples using the standard KBr disk method at frequencies in the range of 400–4400 cm^−1^.

### 2.7. X-ray Diffraction Analysis (XRD)

This analysis was performed in the Department of Chemistry, College of Science, King Saud University. The aim is to study the characteristic diffraction peak shifts of RC powder as a result of its modification by Na ions and CPC after being ground and dried at 50 °C for 48 h. Additionally, the PCNs were analyzed as films. The anode was CuKα radiation, and the wavelength was equivalent to 1.54060 Å with a scanning rate of 3°/min. Furthermore, the scanning range was (3–30 2θ), with a divergence slit of 0.4, while the current and voltage generator was 40.0 mA and 40.0 kV, respectively, at 25 °C.

### 2.8. Transmission Electron Microscopy (TEM)

Transmission Electron Microscopy imaging for the prepared PCNs films was performed in the central laboratory at King Saud University to identify their structure. A small piece of each PCN film was immersed in a suitable resin, then, hardened under high temperature. The diamond knife was then used to cut thin slices of each sample of a thickness of about 70 nm. The slices were then separated by chloroform vapor. Finally, using a thin coated-carbon 200-mesh copper grid held by forceps, the samples were loaded onto the machine. The samples were analyzed under certain conditions with accelerating voltage of 100 kV, and the magnifications were ×100,000 and ×200,000.

### 2.9. Electrochemical Methods

A Potentiostat/Galvanostat ZRA from Gamry Interface 1000 instrument was used. Three methods were performed to evaluate the protection efficiency of C-steel using the prepared coatings according to the following sequence. First, the open circuit potential (OCP) was conducted for 60 min to stabilize the system and reach the steady-state potential (E_SS_) before applying the electrochemical methods. After reaching steady-state potential, electrochemical impedance spectroscopy (EIS) was operated, the sweep range was 10^5^ to 10^−1^ Hz, with an AC amplitude of 10 mV, and 10 points per decade. Second, electrochemical frequency modulation (EFM) was carried out using the frequencies of 2 and 5 Hz, with a base frequency equal to 0.1 Hz; the perturbation amplitude was 10 mV. For EFM, two sine waves at different frequencies were applied to the electrochemical cell simultaneously. The EFM obtained spectrum, the so-called intermodulation spectrum, is a current response as a function of frequency, with the *x*- and *y*-axis representing (frequency) and (current), respectively. Lastly, Potentiodynamic Polarization was performed where the parameters have been set in the Gamry software to align the Tafel conditions. The Tafel plots have been obtained by scanning a potential of ±250 mV according to the potential corrosion value (E_Corr_). All the electrochemical measurements were performed using a three-electrode electrochemical cell, where the working electrode was the C-steel, the reference electrode was the standard calomel electrode (SCE), and the auxiliary/counter electrode was a rigid platinum foil with a surface area of 100 mm^2^ surrounded in a glass body. All the electrodes have been immersed in an aqueous solution of 3.5 wt.% NaCl under room temperature of (20 ± 0.5 °C), open to atmospheric air. Each test was repeated three-to-four times to ensure the validity of the data.

## 3. Results and Discussion

### 3.1. FT-IR Results

#### 3.1.1. FT-IR of the Modified Clay and PCNs

**Figure 1:**
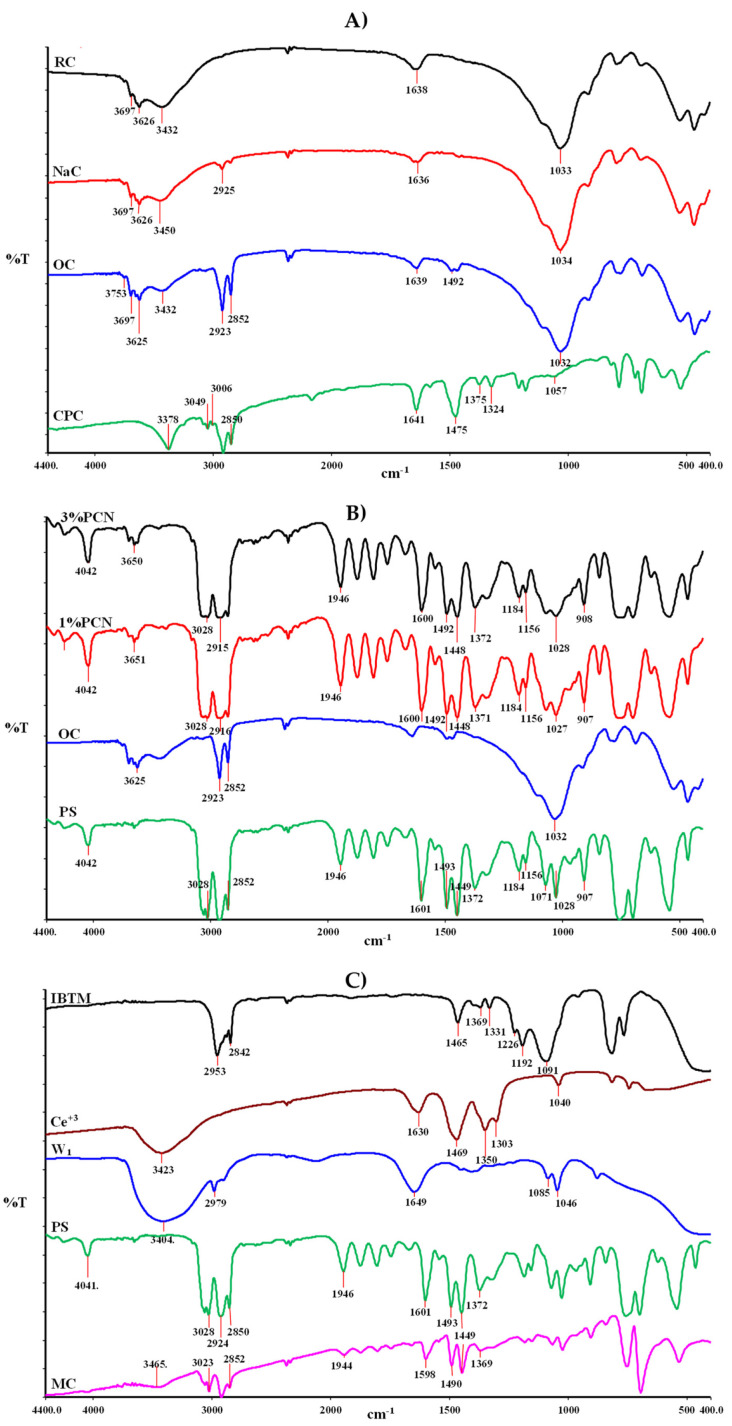
FT-IR of (**A**) RC, NaC, OC and CPC, (**B**) OC, PS, 1% PCN and 3% PCN, and (**C**) PS, Ce^+3^, IBTMS, and MC.

As presented in Figure 1A, the FT-IR spectrum of RC, NaC and OC clearly shows the same characteristic bands. However, the OC shows the characteristic bands of CPC, which indicates the success of the modification process and the transformation of the inorganic NaC into an organic moiety [7,8,9,10,11,13].

The FT-IR spectra of RC, NaC, and OC in Figure 1A show the same characteristic bands of silicates with slight shifts in frequencies. As shown in the clay spectra of RC, NaC and OC, there are absorption bands at the frequencies 3626, 3629, and 3625 cm^−1^, which refer to the stretching vibrations of the (O–H) bonds in Al–OHs, respectively, and conjugated to them the bending vibrations at 915, 916, and 913 cm^−1^, respectively. Medium absorption bands can be seen at frequencies of 1636, 1644 and 1638 cm^−1^, which are the bending vibrations of water molecules in RC, NaC, and OC, respectively. Regarding the absorption bands shown at 1034, 1031, and 1032 cm^−1^, they refer to the stretching vibrations of the (Si–O) bonds in the tetrahedron layers of SiO4 for RC, NaC, and OC, respectively.

Moreover, two bands at frequencies of 2922 and 2852 cm^−1^ refer to the asymmetric and symmetric stretching vibrations of (C–H) bonds of methylene groups (–CH2) in the alkyl chain of CPC. In addition, a new weak band in the OC spectrum at 1195 cm^−1^ may refer to the (C–N) bond stretching from CPC, with a little shift to a lower frequency than the present one in CPC spectrum [7,8,9,10,11,13]. The FTIR spectrum of OC differs from the RC and NaC clay spectra with the presence of additional absorption bands, which proves the success of CPC cations intercalation between the silicate layers (d_001_ spacing), since the absorption band at 3065 cm^−1^ is related to the stretching vibrations of sp^2^ (C–H) bonds from CPC with its bending at 1493 cm^−1^.

In Figure 1B, with regards to the PS spectrum, the two absorption bands appearing at 3061 and 3028 cm^−1^ refer to the aromatic stretching vibration of sp^2^ (C–H). In addition, two strong absorption bands were observed at about 2920 and 2852 cm^−1^, which are related to the asymmetric/symmetric vibrations of the aliphatic (C–H) stretching of(–CH_2_), with its bending vibration at 1449 cm^−1^. It was also noted that the aromatic ring-stretching vibrations of (C=C–C) have absorptions at 1601, 1543, and 1493 cm^−1^, and their several combination or overtone bands can be seen in the range between (1671–1946 cm^−1^). Moreover, three absorption bands at 1071, 1028, and 966 cm^−1^ are related to (in-plane) bending, while the other three bands at 907, 758, and 699 cm^−1^ referred to (out-of-plane) bending of (C–H). A weak absorption band at 4042 cm^−1^ was also noticed; it may suggest the first overtone or combination of stretching vibrations of (C–H) and (C=C–C) bonds in polystyrene [8,9,10,11]. By comparing the infrared spectra of both PS and OC, it is easy to notice the presence of the characteristic absorption bands of PS in the prepared polystyrene clay nanocomposites of 1 wt.% and 3 wt.% PCN, which supports the successful preparation of these nanocomposites by inserting polystyrene chains between the organically modified clay layers (interlayer). With respect to the absorption bands within the spectra of 1% PCN and 3% PCN, the stretching vibrations of the (–OH) of Al–OHs at 3651 and 3650 cm^−1,^ respectively appeared, but their bending vibrations did not appear clearly, probably due to overlapping and (out-of-plane) bending vibrations of (C–H) from PS at the frequency of 907 and 908 cm^−1^ respectively [7,8,9,10,11].

#### 3.1.2. FT-IR of IBTMS, Ce (NO_3_)_3_, W_1_, PS and MCs

As shown in Figure 1C, the spectrum of IBTMS has an absorption band of 2842 cm^−1^, which is attributed to the (C–H) bonds stretching vibrations of methoxy groups (–OCH_3_) in the silane [12]. Also, two sharp absorption bands appeared at 2953 and 2874 cm^−1^ that were assigned to the asymmetric and symmetric stretching vibrations for (C–H) bonds in the methylene group (–CH_2_–). Its bending can be clearly seen as a medium absorption band at 1465 cm^−1^ [12]. The low, intense peaks between 1400 and 1369 cm^−1^ may be due to the deformation vibration of (–CH_2_–) in the methylene groups. A wide, high, and intense absorption band present at 1091 cm^−1^ might suggest the stretching vibration of (Si–O–C) and (Si–O–Si) bonds present in the silane, while the two peaks around 816 and 766 cm^−1^ may be due to (Si–C) and (Si–O) bending modes, respectively [12,14,15]. Regarding the cerium ions spectrum, a strong, broad peak with a frequency of 3423 cm^−1^ is related to the (O–H) stretching vibration of the water molecules, with a bending vibration at about 1630 cm^−1^ [16]. The intense absorption bands at 1469, 1350, 1303, 1040, and 816 cm^−1^ are the typical vibrations associated with the nitrate ions (NO_3_^−1^), so the 1469 and 1350 cm^−1^ bands are related to the asymmetric and symmetric stretching vibrations of (N–O) bonds, respectively, while the absorption band at 1040 cm^−1^ can be attributed to the stretching vibration of free NO_3_^−1^ ions [11,17]. The observed weak peak at a frequency of 550 cm^−1^ corresponds to the stretching vibrations of (Ce–O) [18,19]. When IBTMS and Ce^+3^ spectra are compared, we can easily figure out the characteristic peaks of each of them in the FT-IR spectrum of W_1_. The stretching and bending vibrations of water molecules from cerium ions appeared at 3405 and 1649 cm^−1^, with a slight increase in their broadening and intensities, which may be due to strong chemical bonds. The typical vibrations of nitrates were observed but with fewer intensities in the range of (1455–1376 and 1046) cm^−1^. In contrast, the stretching vibrations of (C–H) bonds related to the methoxy groups from the silanes disappeared, proving that the methoxy groups were completely hydrolyzed during W_1_ preparation. Additionally, a new weak intense band was observed at approximately 878 cm^−1^. This new band is likely associated with (Si–O–Ce) stretching vibrations resulting from the bond formation between the cerium and silicon atoms from the silanols [19,20]. Furthermore, all the absorption bands of PS and W_1_ were observed with little shift toward fewer frequencies, probably due to the conjugation effect, except for the (O–H) stretching vibrations of water molecules, where it was observed at a higher frequency at about 3466 cm^−1^. A weak absorption band at 4042 cm^−1^ was noticed, suggesting the first overtone or combination of stretching vibrations of (C–H) and (C=C–C) bonds in polystyrene [7,13]. The discussed FT-IR spectra of W_1_, PS, and the FT-IR spectrum of the prepared microcapsules (MCs), clearly reveals the success of the encapsulation of W_1_ via PS.

### 3.2. X-ray Diffraction (XRD) Analysis

The XRD analysis is a powerful technique for assessing the intercalations of organic compounds and their arrangements between the layers of smectites and bentonite subgroups. The intercalated organic cations have various configurations and can be identified based on the d_001_ spacing value, as previously reported by Chen et al. [21] and Zhu et al. [22]. The bentonite clay samples, as well as the PS/OC nanocomposites, were analyzed by XRD, and the results are discussed in detail below.

#### 3.2.1. XRD Analysis of RC, NaC and OC

**Figure 2:**
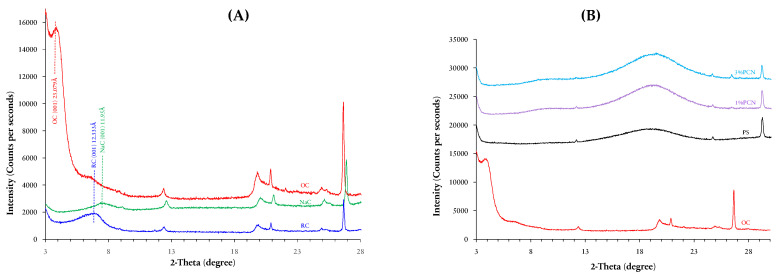
XRD images of (**A**) RC, NaC, OC, and (**B**) OC, PS, 1% PCN, 3% PCN.

The X-ray diffraction analysis was applied in order to confirm the intercalation of the CPC cations into the interlayer according to the (d_001_ spacing) value of the clay’s characteristic peak. After the inorganic modification with NaCl and the organic modification with the surfactant, NaC and OC show the same characteristic peaks as the RC with minor shifts, which proves that the crystalline structure of bentonite clay did not change. The XRD patterns of the RC, NaC, and OC are shown in Figure 2A. The 2θ angle of the (001) reflection corresponding to the basal spacing of the RC is 7.16°, while the d_001_ spacing is 12.33 Å. After the first stage of RC modification of sodium chloride, a decrease in d_001_ spacing was observed to be 11.95 Å in the NaC pattern, and the characteristic diffraction peak shifted slightly to a higher angle of about 7.40°. This shifting may be due to the replacement of the common cations present in the RC interlayer, such as K^+1^ and Ca^+2^, which are known to have a higher radius compared to Na^+1^. In contrast, the diffraction pattern of the OC shows a remarkable increase in the interlayer (d_001_ spacing) up to 23.079 Å, at 2θ equal to 3.825°. This is a confirmation of the CPC cation intercalations into the clay layers and pushing them into a wider distance [8,20,23]. Numerous studies have reported that the basal spacing up to (14 Å) is a monolayer; a bilayer arrangement occurs when the spacing ranged between (14 to 17.7 Å), whereas the spacing value is approximately in the range of (17–22 Å) is a pseudo-trilayer. Finally, a paraffin-type-monolayer or -bilayer usually has a spacing (>22 Å) [24,25,26,27]. From the foregoing discussion, we suggest that the modified organoclay (OC) in our work has two possible configurations: pseudo-tri-layer and paraffin type.

#### 3.2.2. XRD Analysis of PS, 1% PCN and 3% PCN

Further analysis using XRD was applied to OC, PS, 1% and 3% PCN to study the effect of PS chains on OC interlayers using 1 wt.% and 3 wt.% of OC. From the XRD patterns shown in Figure 2B, the PS pattern does not show any diffraction at 2θ from 3° to approximately 14°, but it shows a wide, hill-like diffraction at 2θ in the range of (14°–23°) with low intensity [28,29]. By comparing the previously discussed patterns of OC and PS, we can deduce the effect of PS chain insertion on the layers of OC. In the 1% PCN and 3% PCN patterns, the diffraction peak of polystyrene appeared in the same position. Low crystallinity is the factor behind broad diffraction peaks in PS and PCNs [10]. Importantly, the diffraction peak of the OC at 2θ equal to 3.825° disappeared in the prepared nanocomposites 1% PCN and 3% PCN patterns, which indicates the amorphous PCN structure. This shows the possibility of an exfoliated structure of the prepared PCNs, as found by other researchers [28,29,30]. To support these XRD results, an additional analysis was performed using transmission electron microscopy (TEM).

### 3.3. Scanning Electron Microscopy (SEM)

**Figure 3:**
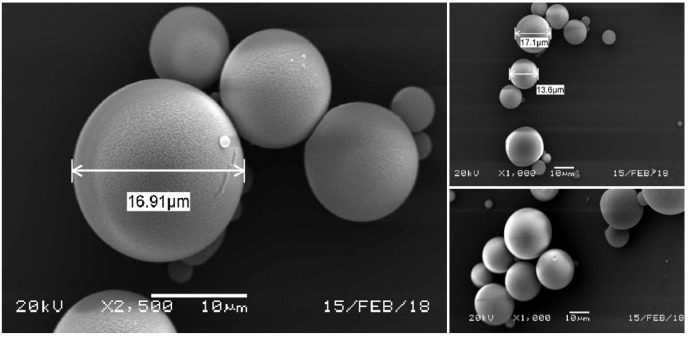
SEM images of the obtained polystyrene microcapsules (MCs).

Based on the preliminary FT-IR results for the prepared polystyrene microcapsules MCs, we can confirm that the MCs were successfully prepared. To verify, SEM analysis was performed to study the other morphological properties. It is worth saying that the MC’s preparation was repeated and when the weight of the starting materials is doubled, the percentage yield increases by about 70%, producing 1.94 g of white MCs with a homogeneous size, which in turn is consistent with the previous studies [31,32]. As shown in the SEM image Figure 3, the perfectly spherical shape of the prepared MCs was obtained with a radius that varies in the range (10–30 µm). In addition, a mononuclear structure was observed with a smooth, nonporous surface, as mentioned by Cotting et al. [5].

### 3.4. Transmission Electron Microscopy (TEM)

**Figure 4:**
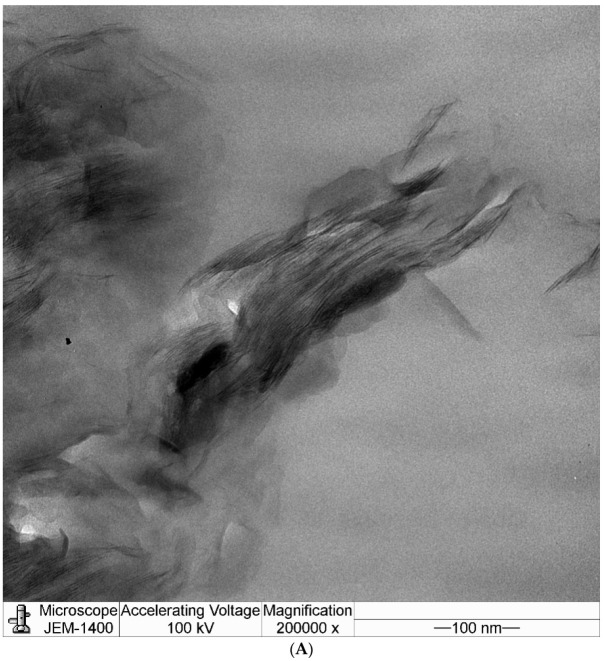

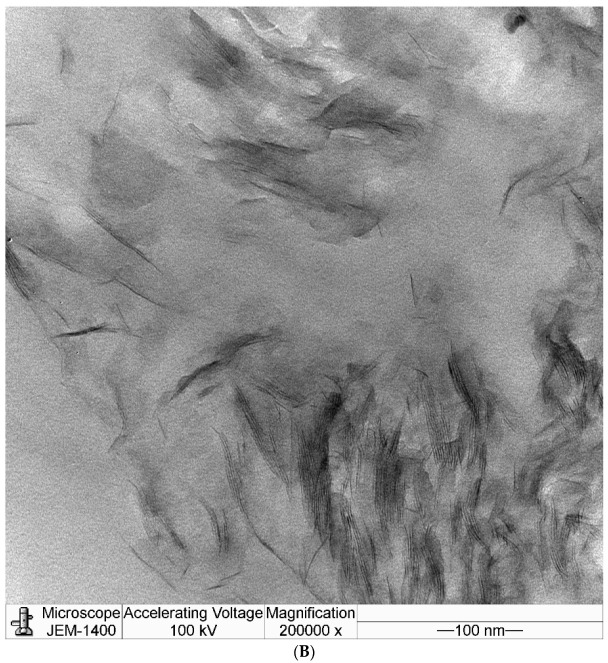
TEM images of (**A**) free 1% PCN at high magnification and (**B**) Free 3% PCN at high magnification.

This technique (TEM) is powerful due to its effectiveness in determining the internal structure of the nanocomposites in the nanoscale range. Thus, the degree of the clay nanolayer exfoliation in polystyrene can be verified. To support the results revealed by FT-IR and XRD, transmission electron microscopy (TEM) analysis was performed on the prepared 1 wt.% and 3 wt.% PCN. The light areas correspond to the polystyrene, while the hair-like dark areas are related to the nanolayers of the clay [28,30]. Both 1% PCN and 3% PCN were imaged at ascending magnifications (100,000 and 200,000). In Figure 4A, the TEM images of 1% PCN are presented. As shown, the clay layers are lined with each other in most areas, maintaining a generally parallel arrangement, indicating the presence of an intercalated structure, while the hair-like areas in the samples provide an exfoliated structure [33,34]. As shown in Figure 4B, free 3% PCN clearly shows that the nanolayers have been totally dispersed throughout the polystyrene matrix. Therefore, combining the XRD and TEM results, it can be observed that the free 3% PCN has an exfoliated structure [35,36]. This result can be strongly observed in the 200,000 magnifications. It is worth noting that the black areas belong to the clay layers that were not dispersed in the polystyrene matrix.

### 3.5. Electrochemical Measurements

In our previous study, [6] The adhesion using the crosscut (cross-hatch) testing for PS and 1, 3, 5, and 10% PCN were determined. The results showed that for the PS, 1% and 5% PCN coatings, the area removed was 35–65%. However, for 3%, none of the coating areas was removed. Thus, 3% PCN provided the best coating adhesion. This improvement in adhesion indicates that the prepared PCN filled the voids and crevices on the C-steel surface. Therefore, in this study, only the low OC loadings were employed. To evaluate the efficiency of the prepared protective coatings, three sequential electrochemical methods were performed as follows: Electrochemical Impedance Spectroscopy (EIS), Electrochemical Frequency Modulation (EFM), then Potentiodynamic polarization. All the measurements were taken after applying Open Circuit Potential (OCP) technique for 1 h, which is the approximate time for systems to attain the steady-state cell potential (E_SS_) as indicated in several studies [37,38,39]. The working electrode (WE) systems are bare and coated C-steel.

#### 3.5.1. Electrochemical Impedance Spectroscopy (EIS)

**Figure 5:**
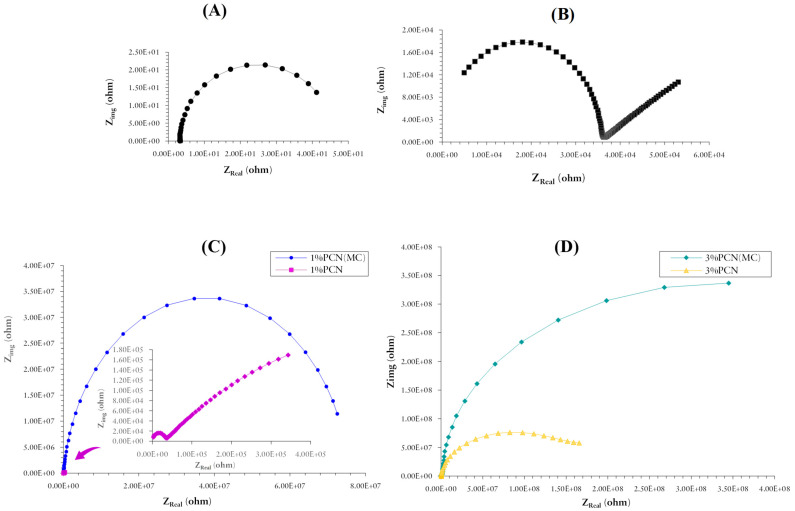
EIS Nyquist plots of (**A**) Bare C-steel, (**B**) PS, (**C**) 1 wt.% PCNs free and impregnated, and (**D**) 3 wt.% PCNs free and impregnated after 1 h of immersion in 3.5 wt.% NaCl at 20 ± 0.5 °C.

During the coating preparation processes, many defects such as micropores, cavities, as well as free volumes are generated in the coating, resulting in penetration of the coating by corrosive electrolytes, causing a reduction in the barrier performance of coating with time [38,39,40]. For the organic coating of metals, the usual interpretation of the impedance diagrams in the high-frequency (HF) part is related to the organic coating, while the low-frequency (LF) part corresponds to the reactions occurring on the metal through defects and pores in the coating [38,39]. In this study, we focus on the Nyquist plots to build our interpretation of EIS data based on their fitted (calculated) data via a suitable equivalent circuit. Two equivalent circuits were used, depending on the corroding system. For the bare C-steel electrode, the simplest circuit known as (Randles) was applied. However, the coated electrode has additional components to compensate for the mechanisms or processes when the coating becomes deformed or penetrated. One of the most effective applications of the EIS has been on the evaluation of the anticorrosive coating’s efficiency and its changes during exposure to corrosive environments. From the Nyquist plot in Figure 5A, we notice only a single semi-circle for the bare C-steel, whereas the Nyquist plots related to the PS-coated samples in Figure 5B and for PCNs free samples in Figure 5C consist of almost two semi-circles. The PCN (MC) in Figure 5B,C show a semi-circle with a larger diameter than the PCN coating, which reflects higher R_corr_ values and higher protection.

**Figure 6:**
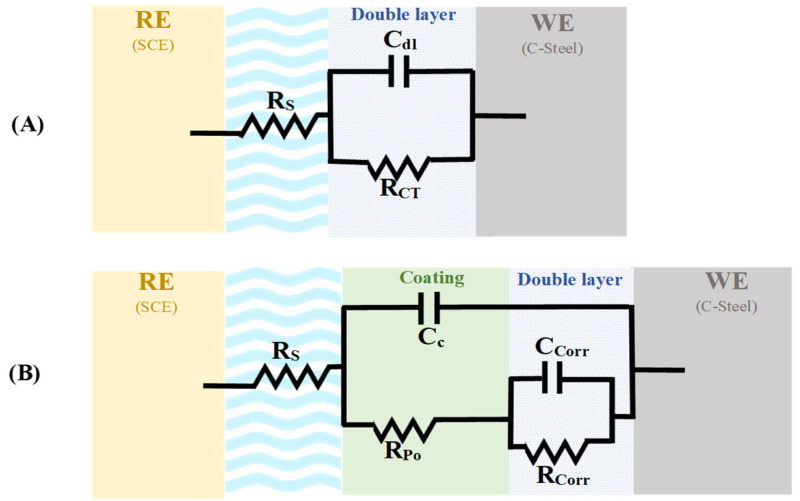
Schematic diagram of the equivalent circuits (**A**) Randles, and (**B**) for free and impregnated PCNs.

The electrical circuit in Figure 6 comprises the electrolyte resistance (R_Sol_), a constant phase element representing the coating capacitance (Cc), the coating resistance to the passage of electrolytes (R_Po_), the constant phase elements representing the double layer capacitance between the metal surface/electrolyte solution (C_Corr_), and the charge transfer resistance across the metal surface (R_Corr_). As reported in the literature for the coated substrates, the first semi-circle in the high-frequency region was related to the resistance and capacitance of the protective coating and its properties [40,41,42]. The second semicircle in the low-frequency region was attributed to the electrochemical reactions on the C-steel surface. Illustrations of the bare and coated C-steel equivalent circuits are shown in Figure 6. These electrical circuits were used for the purpose of fitting the experimental results via the Gamry software. The fitted EIS data of bare C-steel, PS, 1% PCN, 3% PCN, 1% PCN (MC) and 3% PCN (MC) are presented in Table 3. It is observed that Ess increased after adding PCN, compared to the bare C-steel and PS-coated C-steel. Thus, it can be considered that the PCN formulation tend to become more noble (electropositive potential), improving the decrease of the anodic current density compared with the bare metal substrate. It can be noted that the pure polystyrene coating played an important role in the improvement of the protection efficiency of the C-steel compared to bare C-steel. This significant improvement is proven by a comparison between the corrosion resistance R_Corr_ values of bare C-steel, which equals 4.295 × 10^1^ Ω, and that of the C-steel coated with pure PS, which was equal to 2.347 × 10^5^ Ω. This increase in the value of corrosion resistance is coupled with a decrease in the corrosion capacitance C_Corr_ value from 1.332 × 10^−3^ to 5.337 × 10^−5^ F/cm^2^. Regarding the coated C-steel samples with PCNs, which are 1% PCN, 3% PCN, 1% PCN (MC) and 3% PCN (MC), the enhancement of the anticorrosive coating efficiency has also been observed based on the values shown in Table 3, as they were increased almost a thousand time compared to the pure PS. Besides, the effect of raising the weight percentage of the added organoclay from 1 to 3 wt.%, promoted the corrosion resistance as well as reduced the corrosion capacitance related to the double-layer region.

Also, the percentage protection efficiency (%PE) for the coated substrate relative to the pure PS has an average value of approximately 99%, which was estimated using the following equation [43]:

(1) $$\mathrm{PE}\%=[1-(\frac{R_{\mathrm{Corr}}\;\left({}_{\mathrm{PS}}\right)}{R_{\mathrm{Corr}}\;\left({}_{\mathrm{PCN}}\right)})]\;\times \;100$$

Moreover, the capacitance values of either the corrosion or the coating C_Corr_ and C_C,_ respectively, gave a slight change in their values. Overall, this significant improvement in the nanocomposite coating efficiency compared to pure polystyrene is due to the addition of organically modified clay. In more detail, the exfoliated clay layers in PS or the clay nanolayers in PCN, play a significant role in blocking or lengthening the diffusion pathway of the corrosive ions such as chloride and moisture whenever the coating gets damaged or penetrated. This is due to the increased tortuosity of the diffusion path in PCN compared to pure PS coating. Altogether, the coated C-steel by 3% PCN (MC) gave the best protection properties after 1 h of immersion in 3.5 wt.% NaCl, with the highest value of R_Corr_ 6.678 × 10^8^ Ω, with a %PE equivalent to 99.996% compared to the pure PS. This superiority in the protection efficiency of the 3% PCN (MC) sample is likely due to several reasons; the most important is the partially exfoliated structure of the prepared 3 wt.% polystyrene clay nanocomposite that has been verified by TEM results. Also, the new PCN formulation through the addition of polystyrene microcapsules impregnated with Ce^+3^ may have contributed to enhancing the protection efficiency of the coating. The trend in the protection efficiency of the prepared formulations based on the EIS results are in the following order:

3% PCN (MC) > 3% PCN

1% PCN (MC) > 1% PCN

#### 3.5.2. Electrochemical Frequency Modulation (EFM)

As in the EIS technique, electrochemical frequency modulation (EFM) is a non-destructive corrosion measurement technique that can directly give values of the electrochemical corrosion parameters like corrosion current I_Corr_ (expressed in µA) and corrosion rate CR (expressed in mpy) without a prior knowledge of Tafel constants 𝛽a and 𝛽c. In general, the intermodulation spectrum of bare C-steel includes something like peaks going up towards higher current values. The intermodulation spectrum of the coated C-steel showed peaks moving down towards less corrosion current. The electrochemical parameters obtained from EFM measurements of bare C-steel, PS, 1% PCN, 3% PCN, 1% PCN (MC) and 3% PCN (MC) are presented in Table 4. It is noticed that PCNs demonstrate a reduction in the corrosion rate, ending with high efficiency in corrosion protection in comparison with bare substrate. In addition, 3% PCN have provided superior corrosion protection for both free and those impregnated with MCs, 3% PCN (MC). Addition of organically modified clay results in the lengthening of the diffusion pathway of corrosive ions and reducing corrosion current density. According to the obtained corrosion parameters, specifically the corrosion rate (CR) and corrosion current density (I_Corr_) shown in Table 4, the results of the EFM technique coincide with what was obtained from EIS in the reduction of the corrosion rate as well. The measured CR for bare C-steel was 5.365 × 10^1^ mpy, while the CR of other coated C-steel was in the range between 1.583 × 10^−1^ to 1.040 × 10^−5^ mpy. Importantly, the CF(2) and CF(3) are related to the causality factor (2) and causality factor (3), respectively. Based on their experimental values shown in Table 4, they are close to their theoretical standard values, which are equal to (2.00) and (3.00), which in turn, reflects the validity of the calculated CR and I_Corr_ data_._ The deviation of causality factors from their standard values may be due to the perturbation amplitude being too small or the resolution of the frequency spectrum not being high enough, as mentioned in other studies [16,17]. In conclusion, 3% PCN and 3% PCN (MC) samples gave the highest protection efficiency relative to the pure PS with 99.993% and 99.990% PE, respectively. It is worth mentioning that the 3% PCN (MC) is slightly more than 3% PCN, unlike the results of EIS which may be related to method sensitivity.

#### 3.5.3. Potentiodynamic Polarization

Potentiodynamic polarization is a destructive technique of applying DC that provides quantitative information as anodic Tafel constant (𝛽a), cathodic Tafel constant (𝛽c), corrosion current (I_Corr_), corrosion potential (E_Corr_) and corrosion rate (CR) [1,4,44]. The corrosion current density was calculated for each experiment by the intersection of extrapolating the Tafel anodic and cathodic lines at the corrosion potential (E_Corr_). The polarization curves using Tafel plot were collected in the range of (±250 mV vs. E_Corr_). A statistical test was used to estimate how the quality of the obtained measurement data fitted with the expected theoretical results according to the used equivalent circuit, based on its value. In our work, the fitting quality was estimated for each sample by calculating the Chi-squared value. The results reveal a good fitting quality for all the performed measurements, with values ranging between 10^−1^ and 10^−4^. For further clarification, a decrease in the Chi-squared value resulted in an increase in the fitting quality, as reported by Lim et al. [45]. 

**Figure 7:**
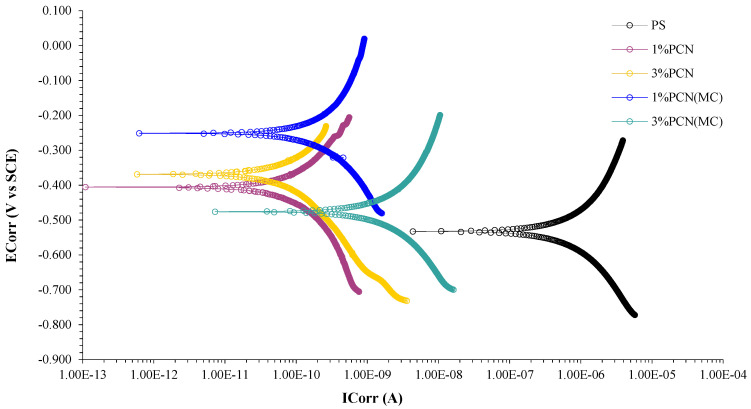
Tafel plots of PS, 1% PCN, 3% PCN, 1% PCN (MC) and 3% PCN (MC) after 1 h of immersion in 3.5 wt.% NaCl at room temperature 20 ± 0.5 °C.

Initially, we noticed that the corrosion potential (E_Corr_) values of the coated C-steel shifted slightly towards more positive values compared to the corrosion potential of bare C-steel, which has a value of −634 mV. This can also be seen when C- steel coated with polystyrene is compared to that coated with PCNs formulation, whether free or impregnated, as shown in Figure 7. This behavior has been discussed in previous studies, proving the efficient protection of the prepared PCN coatings [46,47]. The test specimen 3% PCN provided the best corrosion resistance with a corrosion rate equal to 4.128 × 10^−6^ mpy, and corrosion current (I_Corr_) equivalent to 4.290 × 10^−5^ µA after 1 h of immersion. The calculated protection efficiency percentage of 3% PCN relative to the pure PS was the highest, with percentages of about 99.998%. The obtained Potentiodynamic polarization parameters of bare C-steel, PS and coated PCNs are presented in Table 5. The obtained results from these three electrochemical methods after 1 h of immersion are not sufficient to evaluate the enhanced efficiency of the impregnated coatings over time; we therefore studied their efficiency over time using the EIS nondestructive method.

#### 3.5.4. Evaluation of the Mechanism of Enhanced Coating Protection

**Scheme 2:**
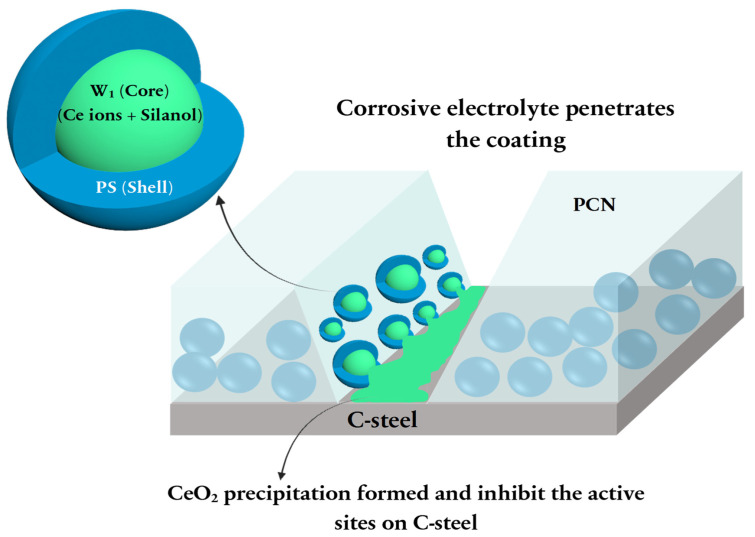
The role of microcapsules in enhancing the efficiency Coating.

Evaluating the healing performance of smart coatings has no standards. There is no guidance on the types of experimental methods that will provide consistent information for understanding the exact process or mechanism of self-healing. Numerous previous studies used the electrochemical impedance spectroscopy (EIS) technique to study the self-healing behavior of protective coatings to confirm the role of MCs on the prepared nanocomposite coating [48,49,50,51,52]. Thus, EIS measurements were set up for both 1 wt.% and 3 wt.% PCNs with free and impregnated PCN coatings. The measurements were performed during exposure to the corrosive electrolyte 3.5 wt.% NaCl at room temperature 20 ± 0.5 °C, after 1, 4, 24, and 48 h. Once the electrolyte diffuses through the coating, an electrochemical corrosion reaction takes place on the non-adherent area of the C-steel. This leads to C-steel dissolution accompanied by a reduction reaction of the dissolved oxygen in the solution. The corrosion product is ferric hydroxide with a formula of Fe(OH)_3_. As a result of coating penetration, healing agents stored inside MCs will diffuse [5,49]. Thus, the self-healing mechanism may consist of two processes. The silanol functionality relies on its coupling agent property as well as forming a non-soluble adsorbed film on the surface of the C-steel electrode. Condensation of silanol may occur, followed by hydrogen bonding formation with the hydroxyl groups present on the surface of the C-steel. This mechanism finishes with a decrease in the corrosion reaction via adsorption as well as increasing the coating adhesion. The trivalent cerium ions may possibly undergo a series of chemical reactions, depending on the present system components. This is depicted in Scheme 2. Danaee et al. [49] suggested the oxidation reaction of the Ce^+3^ ions to form cerium hydroxide Ce(OH)_3_. Thereafter, further reactions, including precipitation, might result in the formation of cerium oxide CeO_2_. Suggested chemical reactions of trivalent cerium ions are shown below [49]:

2H_2_O + O_2_ + 4e ⟶ 4OH^−^ (2)

Ce^+3^(aq) + 3(OH^−^) ⟶ Ce(OH)_3_ (3)

2Ce(OH)_3_ +2 (OH^−^) ⟶ 2CeO_2_(s) + 2H_2_O + 2e (4)

**Figure 8:**
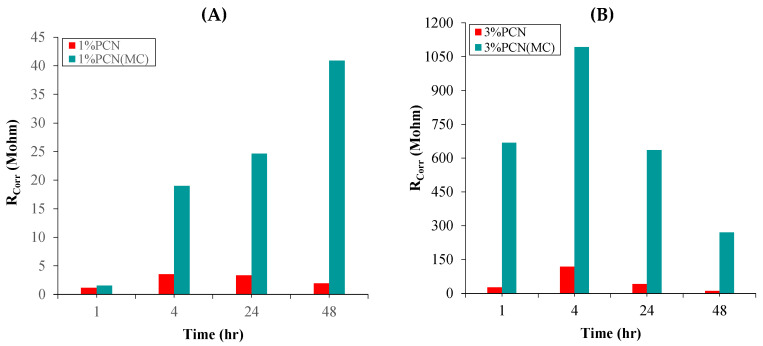
Corrosion resistance R_Corr_ (MΩ) vs. time at 1, 4, 24 and 48 h for (**A**) Free and impregnated 1%PCNs, and (**B**) Free and impregnated 3%PCNs immersed in 3.5 wt.% NaCl at room temperature 20 ± 0.5 °C.

The deposition of cerium oxides and hydroxides on the ferric hydroxide layer blocks the cracks and causes the “self-healing” ability. Consequently, the coating is healed and adherent to the C-steel substrate by the efforts of both the adsorbed silanol and CeO_2_. As a result, the protection efficiency will be enhanced to provide not only a barrier property but long-term protection. From our study, the best evidence for this mechanism is the evaluation of the electrochemical parameters from the EIS measurements after 48 h of immersion. The obtained EIS fitted data for both 1 wt.% and 3 wt.% PCNs free and impregnated are summarized in Table 6 and shown in Figure 8. During the time of exposure, it was observed that the steady-state potential (E_SS_) shifted towards more positive values, indicating a strengthening in the protective film. One of the most important parameters for assessing the coating integrity is the (C_C_) values. Unlike the expected, the impregnated coating capacitance of 1% PCN (MC) decreased significantly from its initial value 3.885 × 10^−10^ F.cm^−2^ towards lesser values as follows: 1.628 × 10^−10^, 1.467 × 10^−10^ and 1.562 × 10^−10^ F.cm^−2^ during immersion times of 4–48 h. These changes in decreasing coating capacitance values can be attributed to the diffusion of the electrolyte through the coating as well as the MCs shell, leading to the release and diffusion of corrosion inhibitors. Pore resistance (R_Po_) of 1% PCN (MC) after 4 h of immersion was reduced from 74.1 MΩ to 8.839 MΩ which was associated with C_C_ reduction as well. Moreover, after 24 h, a noticeable increment in the pore resistance to a value of 11.53 MΩ was noticed, reflecting that the coating may have undergone a healing process by blocking the electrolyte diffusion pathways with the formation of CeO_2_. In addition, the increase in corrosion resistance (R_Corr_) was noted, in addition to ascending behavior during the time of exposure. It is shown in Figure 8 that the effectiveness of 1% PCN (MC) continued even after 48 h of exposure to the electrolyte with the inhibitor’s releasing process. The same argument applies to 3% PCN (MC) but with higher corrosion parameters, which may be due to the exfoliated structure, as shown from TEM results in Figure 4. It is worth mentioning that although the 3% PCN (MC) has a higher R_corr_ at each time interval, it reached its highest R_corr_ after 4 h then gradually decreased with time. These results are consistent with the results of previous studies on the self-healing performance in protective coatings [5,49,50,51,52,53]. In conclusion, the enhanced properties of the developed PCN (MC) coatings make them attractive for their potential application in the oil industry and many industrial applications.

## 4. Conclusions

Extending our previous findings that low organic clay loading gave the best coating protection, only 1% and 3% PCN formulations were investigated in this study. Moreover, new protective coatings were prepared from polystyrene/organoclay nanocomposites (PCNs) impregnated with polystyrene microcapsules (MCs) loaded with inhibitors (Ce^+3^ ions and silanol), labeled as 1% PCN (MC) and 3% PCN (MC). The Structural and morphological characterization techniques confirmed the success of preparing the new coating formulations. The electrochemical measurements confirmed the enhancement in the protection efficiency of PCN (MC) compared with the PS-coated samples and the unimpregnated samples. The prepared, 3% PCN(MC clearly exhibited more efficient protection properties reaching 270.5 MΩ after 48 h of immersion, higher than all the other formulations. The diffusion mechanism of the inhibitors released from the MCs has been suggested through CeO_2_ precipitation on the C-steel surface after being released and by the silanol functionality as a coupling agent. The enhanced properties of the developed PCN (MC) coatings make them attractive for their potential application in the oil industry.

## Figures and Tables

**Table 1:** The chemical composition of the carbon steel (C-steel).

Element	C	Mn	Cr	Si	Ni	Cu	Al	P	S
**Weight (%)**	0.46	0.6	0.18	0.18	0.04	0.03	0.023	0.013	0.006

**Table 2:** The material used in this work.

Product Name	Source
RC	Khulays clay—Saudi Arabia
C-steel (Grade 1046)	ODS Co.—Berlin, Germany
Polystyrene (PS125)	SABIC—Riyadh, Saudi Arabia
Sodium Chloride (AR grade)	Win lab Company, Queensland, Australia
Ethanol 96%—AR grade	Avonchem—Waterloo, UK
Cetyl pyridinium chloride (CPC)	BDH Co., Istanbul, Turkey
Polyvinyl Alcohol (PVA)	LOBAChemie Co., Mumbai, India
Glacial acetic acid 99.5%—Extra Pure	Sigma-Aldrich Co., Saint Louis, MI, USA
Dichloromethane 99.5%—AR\ACS grade	Sigma-Aldrich Co.
Isobutyl Trimethoxy Silane 97% (IBTMS)	Alfa Aeasar Co., Ward Hill, MA, USA
Ce(NO_3_)_3_.6H_2_O—AR	LOBAChemie Co.

**Table 3:** The EIS fitted data of bare C-steel, PS, 1% PCN, 3% PCN, 1% PCN (MC) and 3% PCN (MC) after 1 h of immersion in 3.5 wt.% NaCl at room temperature 20 ± 0.5 °C.

Sample	E_SS_ (mv)	R_Corr_ (MΩ)	R_Po_ (MΩ)	C_Corr_ (nF/cm^2^)	CC (nF/cm^2^)	% PE
**Bare (C-steel)**	−601	4.30 × 10^−5^	-	1.332 × 10^6^	-	-
**PS**	−549	0.235	0.0555	5.337 × 10^4^	0.1108	-
**1% PCN**	−455	1.181	0.311	2.589 × 10^3^	0.1857	80.127
**1% PCN (MC)**	−215	1.556	74.10	1.022	0.3885	84.916
**3% PCN**	−480	27.52	6.16	0.298	0.1430	99.146
**3% PCN (MC)**	−297	667.8	8.191	0.1142	0.1988	99.964

**Table 4:** The EFM results of bare C-steel, PS, 1% PCN, 3% PCN, 1% PCN (MC) and 3% PCN (MC) after (1 h) of immersion in 3.5 wt.% NaCl at room temperature 20 ± 0.5 °C.

Sample	I_Corr_ (µA)	CR (mpy)	CF(2)	CF(3)	PE%
**Bare (C-steel)**	5.577 × 10^2^	5.365 × 10^1^	2.132	2.694	-
**PS**	1.645	1.583 × 10^−1^	1.095	2.416	-
**1% PCN**	3.697 × 10^−4^	3.556 × 10^−5^	1.469	1.455	99.977
**1% PCN (MC)**	5.553 × 10^−4^	5.341 × 10^−5^	2.101	3.331	99.966
**3% PCN**	1.081 × 10^−4^	1.040 × 10^−5^	1.627	1.043	99.993
**3% PCN (MC)**	1.579 × 10^−4^	1.519 × 10^−5^	1.743	2.689	99.990

**Table 5:** Tafel parameters of bare C-steel, PS, 1% PCN, 3% PCN, 1% PCN (MC) and 3% PCN (MC) after (1 h) of immersion in 3.5 wt.% NaCl at room temperature 20 ± 0.5 °C.

Sample	E_Corr_ (mV)	I_Corr_ (µA)	CR (mpy)	Chi.Sq.	PE%
**Bare (C-steel)**	−634	5.61× 10^1^	5.397	6.359	-
**PS**	−532	2.660	2.557 × 10^−1^	1.8 × 10^−1^	-
**1% PCN**	−404	2.670 × 10^−4^	2.569 × 10^−5^	6.61 × 10^−4^	99.989
**1% PCN (MC)**	−251	1.820 × 10^−3^	1.754 × 10^−4^	1.1 × 10^−3^	99.931
**3% PCN**	−369	4.290 × 10^−5^	4.128 × 10^−6^	2.48 × 10^−2^	99.998
**3% PCN (MC)**	−478	5.820 × 10^−3^	5.602 × 10^−4^	1.52 × 10^−3^	99.781

**Table 6:** EIS fitted data of 1% PCN, 3% PCN, 1% PCN (MC) and 3% PCN (MC) after immersion in 3.5 wt.% NaCl for 1, 4, 24, and 48 h at room temperature 20 ± 0.5 °C.

Sample	Time (Hour)	R_Corr_ (MΩ)	R_Po_ (MΩ)	C_Corr_ (nF.cm^−2^)	C_C_ (nF.cm^−2^)
**1% PCN**	1	1.181	0.3108	2.584 × 10^3^	0.1857
	4	3.536	0.5156	3.725 × 10^3^	0.1399
	24	3.354	3.122	172.2	0.1493
	48	1.936	3.295	285.1	0.1624
**1% PCN(MC)**	1	1.556	74.10	1.022	0.3885
	4	18.97	8.839	0.4099	0.1623
	24	24.64	11.53	0.3899	0.1467
	48	40.91	7.013	0.1520	0.1562
**3% PCN**	1	27.52	6.16	0.298	0.1430
	4	118.6	42.65	0.1067	0.1152
	24	42.06	18.58	0.1791	0.1526
	48	11.41	3.514	24.05	0.1497
**3% PCN(MC)**	1	667.8	8.191	0.1142	0.1988
	4	1092	17.03	0.0113	0.3412
	24	636.1	8.951	0.1682	0.2586
	48	270.5	7.109	0.1635	0.1221

## Data Availability

Some of the raw/processed data required to reproduce these findings cannot be shared at this time as the data also forms part of an ongoing study.
